# Urodynamic function during sleep-like brain states in urethane anesthetized rats

**DOI:** 10.1016/j.neuroscience.2015.11.027

**Published:** 2016-01-28

**Authors:** J. Crook, T. Lovick

**Affiliations:** Physiology and Pharmacology, University of Bristol, Bristol BS8 1TD, UK

**Keywords:** EEG, electroencephalogram, EMG, electromyogram, EUS, external urethral sphincter, PAG, periaqueductal gray matter, REM, rapid eye movement, SEM, standard error of the mean, STFT, short-term Fourier transform, SWA, slow wave activity, vlPAG, ventrolateral periaqueductal gray matter, micturition, urethane, sleep-like EEG state, periaqueductal gray, rat, bladder urodynamics

## Abstract

The aim was to investigate urodynamic parameters and functional excitability of the periaqueductal gray matter (PAG) during changes in sleep-like brain states in urethane anesthetized rats. Simultaneous recordings of detrusor pressure, external urethral sphincter (EUS) electromyogram (EMG), cortical electroencephalogram (EEG), and single-unit activity in the PAG were made during repeated voiding induced by continuous infusion of saline into the bladder.

The EEG cycled between synchronized, high-amplitude slow wave activity (SWA) and desynchronized low-amplitude fast activity similar to slow wave and ‘activated’ sleep-like brain states. During (SWA, 0.5–1.5 Hz synchronized oscillation of the EEG waveform) voiding became more irregular than in the ‘activated’ brain state (2–5 Hz low-amplitude desynchronized EEG waveform) and detrusor void pressure threshold, void volume threshold and the duration of bursting activity in the external urethral sphincter EMG were raised. The spontaneous firing rate of 23/52 neurons recorded within the caudal PAG and adjacent tegmentum was linked to the EEG state, with the majority of responsive cells (92%) firing more slowly during SWA. Almost a quarter of the cells recorded (12/52) showed phasic changes in firing rate that were linked to the occurrence of voids. Inhibition (*n* = 6), excitation (*n* = 4) or excitation/inhibition (*n* = 2) was seen. The spontaneous firing rate of 83% of the micturition-responsive cells was sensitive to changes in EEG state. In nine of the 12 responsive cells (75%) the responses were reduced during SWA.

We propose that during different sleep-like brain states changes in urodynamic properties occur which may be linked to changing excitability of the micturition circuitry in the periaqueductal gray.

## Highlights

• Spontaneous changes in sleep-like EEG state occur in urethane-anesthetized rats.
• The micturition threshold is increased during the slow wave sleep-like state.
• Micturition-related activity of PAG neurons was reduced during slow wave EEG states.
• Changes in sleep-like EEG state may impact PAG-mediated control of bodily systems.

## Introduction

During the sleeping period urine production normally decreases compared to the waking state. This is due in part to the absence of fluid intake, coupled with a nocturnal increase in secretion of antidiuretic hormone. The storage capacity of the bladder also increases due to functional changes, for example in gap junctions in bladder smooth muscle cells, associated with a diurnal change in expression of connexin43 (Negoro et al., 2012, Negoro et al., 2013). During a sleeping period humans typically cycle through a number of sleep stages. These are defined by characteristic changes in the electroencephalogram (EEG) waveform, which cycles between low frequency, synchronized high-amplitude activity (slow wave sleep) and high frequency, desynchronized, low-amplitude activity known as desynchronized or rapid eye movement (REM) sleep. Little is known about the functional activity of the central micturition control circuitry during different sleep states and how this might impact on control of bladder function. Rats, like humans, show a diurnal pattern in sleep and wakefulness (although this is reversed with respect to their light–dark cycle) and different sleep stages can be identified according to characteristic patterns of the EEG waveform (Gottesmann, 1992). Under urethane anesthesia rats show spontaneous changes in the EEG that resemble slow wave and REM brain sleep states seen in unanesthetized animals (Clement et al., 2008). It is thought that urethane promotes unconsciousness by exploiting the brain mechanisms involved in natural sleep, unlike other general anesthetics that induce a pharmacological unitary sleep-like slow wave EEG state (Clement et al., 2008). The urethane-anesthetized preparation has therefore been used as a model to investigate central mechanisms underlying sleep. Another feature of urethane anesthesia is that the micturition reflex is preserved, unlike other commonly used anesthetics (Yoshiyama et al., 2013). Thus this preparation offers the advantage of investigating urodynamics and basic central control mechanisms of micturition during different sleep-like brain states more easily than in chronically instrumented, unanesthetized animals. In the present study we used this preparation as a model system to facilitate mechanistic studies into control of micturition in different sleep-like brain states.

Micturition is dependent on the functional integrity of a spino-midbrain-spinal loop, which relays in the caudal ventrolateral periaqueductal gray matter (vlPAG) (Stone et al., 2011). The vlPAG is thought to act as a neural switch which, in the conscious animal, is under the control of higher centers that can switch the circuitry between storage and voiding modes (see de Groat et al., 2015 for a recent comprehensive review). We carried out continuous cystometry while simultaneously making urodynamic measurements and monitoring activity in the micturition control circuitry in the PAG.

## Experimental procedures

The study conforms to the national guidelines for the care and use of animals and was carried out under the authority of UK Home Office Project Licence PPL30/3200.

29 Male Wistar rats (270–450 g) were used for the study. They were anesthetized with urethane (1.4 g kg^−1^ i.p.) and instrumented to record cortical EEG, femoral arterial blood pressure, heart rate and respiratory airflow. The trachea was cannulated to maintain a patent airway and a femoral venous catheter was inserted for infusion of fluids; rectal temperature was maintained at 37 °C by a homeothermic blanket system (Harvard Apparatus, Holliston, Massachusetts, USA). Following the preparatory surgery, animals were positioned in a stereotaxic frame and a craniotomy was performed to give access to the left periaqueductal gray matter (PAG). The lower body was rotated to allow access to the abdomen. Following a midline laparotomy to expose the bladder, a 25-G needle tip attached to a length of saline-filled polythene tubing was inserted through the bladder dome. A T-piece in the line enabled simultaneous recording of intravesical pressure while infusing saline into the bladder. Two insulated needle electrodes were inserted into the external urethral sphincter (EUS) muscle to record electromyogram (EMG) activity. The time and volume of voids evoked by infusing saline into the bladder was measured either by a drop counter positioned to register the passage of drops of urine falling from the penis or by collecting urine in a beaker placed on the platform of an electronic balance with output to the data acquisition system. All data were captured and displayed using a PowerLab 8SP running Chart v5 software, and exported to Matlab R1013a for further analysis.

### Neuronal recording

#### Single units

In six rats activity of single neurons within the vlPAG and surrounding tegmentum during voiding was recorded with a single-channel electrode. The dura mater was removed from the cortical surface and a parylene coated tungsten microelectrode (A-M Systems Inc, Carlsborg, Washington, USA) was lowered into the PAG. Single-unit activity was amplified (5000×) and bandpass filtered (300–5000 Hz) using a Neurolog system (Digitimer Ltd, Welwyn Garden City, Hertfordshire, UK). If necessary, residual electrical interference was eliminated using a Hum Bug device (Quest Scientific, North Vancouver, British Columbia, Canada). Activity was digitally sampled at 20 kHz and sorted offline by a computer running Spike2 (v7) software.

#### Multichannel recording

In 14 animals, neuronal activity in the PAG was sampled using silicon probes (10-mm shank length, 50-μm probe thickness, width 1.25–1.48 mm with a bevelled end, Poly3 and Edge designs) with 10–32 active channels (NeuroNexus, Ann Arbor, Michigan, USA). Activity was amplified and digitized with a PZ2 Preamplifier (Tucker Davis Technologies, Alachua, Florida, USA) and bandpass filtered (300–5000 Hz) using a RZ5D processor (Tucker Davis Technologies, USA). Spike sorting was carried out offline using KlustKwik (Ken Harris, Rutgers University, Newark, NJ) with evaluation and correction as required using MClust (A. David Redish, University of Minnesota, Minneapolis MN). In all experiments microelectrodes and silicon probes were coated with DiI (50 mg ml^−1^ in ethanol, Biotium, Hayward, California, USA) and allowed to dry prior to insertion into the brain. Fluorescent electrode tracks were later located in histological material.

### Experimental protocol

Once the surgical preparation was completed, a period of 30 min was allowed before starting continuous infusion of saline into the bladder (6 ml h^−1^). Once repeated cycles of filling and voiding had established, the recording electrode was inserted into the brain at approximately 7.3–7.8 mm caudal and 0.8–1.2 mm lateral to the bregma. To target the PAG the electrode was angled 0–10° caudally and 0–5° medially and advanced so the tip was 5 mm below the cortical surface. The electrode was then moved slowly within the target area until single unit spiking activity was clearly distinguishable on the single-channel electrode or on at least one site on the multichannel probes. Continuous recordings of unit activity were carried out over periods of up to 3 h.

### Analysis of EEG waveform

**Fig. 1:**
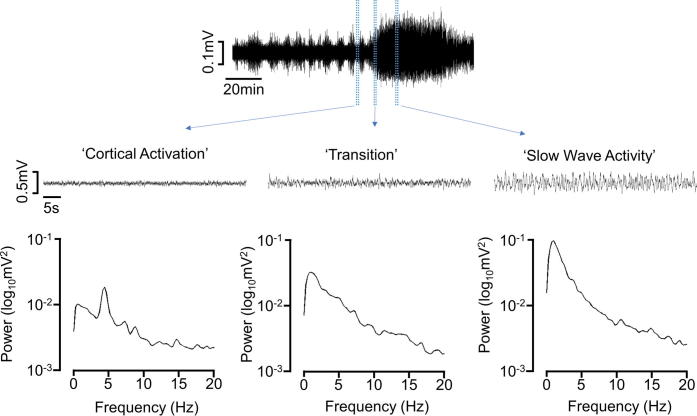
Top row; example of EEG cycling from low-amplitude activated state to slow wave activity. Excerpts show activated, transitional, and slow wave activity on a faster time scale. Bottom row: power spectra of EEG during activated state, transitional, and slow wave activity.

For each animal, analysis of urodynamic and EEG state was conducted over a 2-h period after the micturition reflex had been reliably established. The EEG state was quantified by measuring the power in the 0.5–1.5-Hz bandwidth of the EEG signal using a short term Fourier transform (STFT), with a window length of 2.025 s, and a hop size of 0.5 s. The ‘slow wave power’ was obtained by calculating the mean power in the 0.5–1.5-Hz band and smoothing using a moving average over 25 s (Fig 1). Slow wave power values at the time of voiding were divided into upper and lower quartiles and used to assign voids to ‘slow wave activity (SWA)’ and ‘Cortical Activation’ brain states, respectively.

### Analysis of urodynamic data

For each void, the following measurements were obtained:

#### Void pressure threshold

The pressure within the bladder at the end of the filling phase, prior to the sharp pressure rise at the onset of a void.

#### Void volume and voiding volume threshold

The volume of fluid expelled per void was assessed from the weight of the fluid expelled, assuming a specific gravity of one. Relative changes in voiding volume threshold were derived from the filling rate and void volume measurements.

#### Start and end fill compliance

**Fig. 2:**
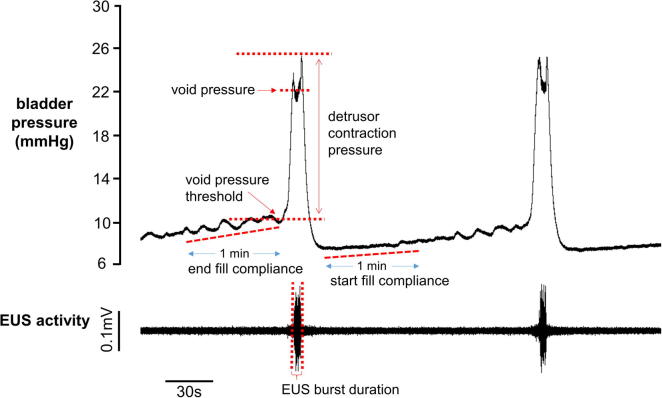
Bladder pressure and external urethral sphincter (EUS) EMG activity during voids.

End-fill and start-fill compliance were measured over a 1-min period prior to and immediately following voiding, respectively (Fig 2). A linear fit was applied to the bladder pressure trace over these periods, and the compliance calculated as the inverse of the pressure rise. Compliance values were discarded for voids with an inter-void interval shorter than 1 min, or for those where the slope of the rise in bladder pressure could not be accurately determined due to the presence of large non-voiding contractions (a sharp rise in bladder pressure not accompanied by bursting activity of the EUS EMG or urine release).

#### Detrusor contraction pressure

Detrusor contraction pressure was defined as the difference between the maximum bladder pressure reached during voiding detrusor contractions, and the void pressure threshold (Fig 2).

#### Void pressure

All voids were accompanied by one or more discrete periods of bursting activity in the EUS EMG, during which time urine was expelled from the urethra. Void pressure was defined as the mean bladder pressure observed during these periods.

#### Void interval

The void interval was defined as the amount of time elapsed since the previous void.

#### EUS EMG, burst duration, number of bursting episodes

The number and total duration of EUS EMG bursting episodes were manually measured for each void.

### Analysis of single unit activity

#### Spontaneous firing

For each unit, mean firing frequency was calculated between the times of each EEG STFT hop (0.5-s interval), and smoothed using a moving average over 25 s. Activity between voids and non-voiding contractions, with a 5-s margin, was considered ‘spontaneous firing’. Spontaneous firing rates at top and bottom quartiles of slow wave power (see 2.3 – Analysis of EEG waveform) were taken as measures of baseline firing for ‘slow wave’ and ‘cortical activation’ brain states, respectively. Spearman’s rank correlation was carried out between slow wave power and spontaneous firing rate. A Spearman’s *r* value <−0.5 or >0.5, and a *p* value <0.05, was considered to reflect a physiologically and statistically significant correlation.

#### Micturition-linked activity

In order to identify micturition responsive cells, raster plots were generated by synchronizing sequential void-associated recordings to the start of the initial bladder pressure rise. Mean and the standard error of the mean (SEM) of firing rate across all voids was calculated using 1-s time bins.

### Localization of recording sites

At the end of the experiment, animals were killed with an overdose of anesthetic and the brains were dissected out and fixed with 4% paraformaldehyde in 0.1 M phosphate buffer for at least 24 h*.* Brains were then cryoprotected by immersion in 30% sucrose in phosphate buffer until they sank (typically 1–2 days). Coronal sections of midbrain (100-μm thick) were cut on a freezing-microtome. Sections were mounted on glass slides and viewed with a fluorescence microscope (Zeiss, Cambridge, UK) under green light (565-nm wavelength) in order visualize electrode tracks in the tissue.

### Statistical analysis

Statistical analyses were carried out using GraphPad Prism v5 software. Paired T-tests were carried out in order to compare urodynamic measurements obtained in ‘activated’ and slow wave EEG states. D’Agostino–Pearson omnibus tests were carried out in order to assess whether the magnitudes of changes in urodynamic measurements were normally distributed across animals. Compliance measurements were log_10_ transformed prior to statistical analysis. The threshold for statistical significance was defined as *p* < 0.05. All values are quoted in text as mean ± SEM, unless otherwise stated.

## Results

### Bladder cystometry

**Fig. 3:**
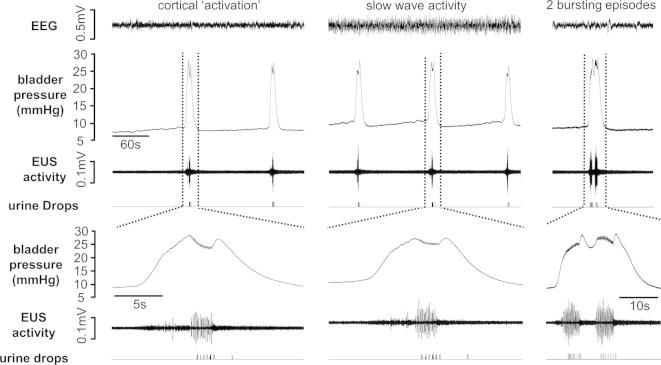
Examples of changes in bladder pressure and activity in the external urethral sphincter (EUS) during repeated voids evoked by continuous infusion of 165 mM saline into the bladder during activated (left panel) and slow wave (middle panel) activity brain states. Right hand panel shows double voids, which occurred more frequently during activated sleep-like activity.

In 20 rats (69% of animals tested) continuous infusion of saline into the bladder (6 ml h^−1^) evoked repeated cycles of filling and voiding with a period of 4.7 ± 0.38 min (Fig 3). Once established, voiding usually continued uninterrupted for 5–10 h. The micturition reflex failed to establish In 11 animals (31%). In these rats bladder pressure rose during the infusion of saline until the animal entered a state of “overflow incontinence” during which bladder pressure remained high and fluid dripped from the penis at regular intervals.

Of the 20 animals that displayed regular voiding, data from two rats were excluded from urodynamic analysis, due to the occurrence of frequent large non-voiding bladder contractions, which precluded accurate urodynamic measurement. In the remainder (*n* = 18), resting bladder pressure rose gradually during the filling period. At the onset of a void the pressure rose sharply. At the same time tonic EMG activity in the EUS also increased and then switched abruptly to bursting activity (7 Hz), each burst lasting for 3.1 ± 0.38 s (Fig 3). Two or more discrete bursting episodes often occurred during a void (Fig 3), although the incidence of these varied greatly between animals. Overall 56 ± 34% (standard deviation) of voids were associated with multiple bursts in EMG activity from the EUS. During the bursting period several drops of urine were expelled from the penis; thereafter bladder pressure fell back to a baseline level and tonic activity resumed in the EUS EMG, decreasing to a baseline value over the next 15–163 s (median 52 s) before the cycle repeated itself (Fig 3).

### Changes in EEG waveform

Once the surgical preparation of the animal had been completed, the EEG typically displayed high-amplitude synchronized activity. Over a period of time, which varied considerably between animals, cyclical changes in the EEG waveform developed and the SWA became interspersed with periods of low-amplitude desynchronized activity with a period of 4–158 min, median 47 min. The synchronized high-amplitude, low-frequency (1 Hz) activity, referred to hereafter as SWA, was replaced by low-amplitude, high-frequency (3–5 Hz) desynchronized activity, which resembled the activated state of REM sleep (Clement et al., 2008) (Fig 3). Cyclical activity in the EEG failed to develop in two animals; data from these experiments were excluded from further analysis.

In rats that showed cyclical changes in EEG activity, transition from the activated brain state to SWA was accompanied by a small increase in mean arterial pressure (95.8 ± 9.0 mmHg to 100.6 ± 9.6 mmHg, *p* < 0.01, paired t-test) and a decrease in respiration rate from 117 ± 9 breaths min^−1^ to 108 ± 12 breaths min^−1^ (*p* < 0.001). Animals also displayed fewer augmented breaths during SWA (0.56 ± 0.23 breaths min^−1^ to 0.19 ± 0.11 breaths min^−1^, *p* < 0.01). No change in heart rate was observed (439 ± 54 bpm *vs.* 436 ± 52 bpm). The transition from one brain state to the other was not accompanied by any obvious change in depth of anesthesia as judged from the pedal reflex and cardiorespiratory measurements. Indeed, the anesthetic level appeared constant over the 8–10-h experimental period and supplementary doses of urethane were required only in some animals toward the end of the experiment. In five animals (35.7%), the initial rise in bladder pressure at the onset of the void was associated with a short-lasting (6.1 ± 4.3 s) switch to low-amplitude, high-frequency desynchronized activity. Such “microarousals” accompanied 8.8% of voids during SWA, and 9.5% of voids during transitions between EEG states. No change was observed during ‘activated’ EEG brain states.

### Urodynamic measurements during changes in EEG brain state

**Fig. 4:**
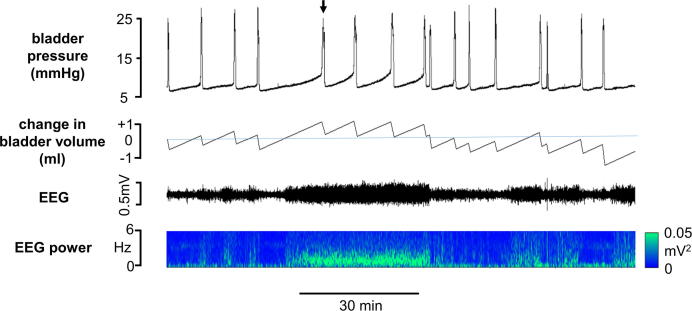
Change in urodynamic parameters of voids evoked by continuous infusion of saline into the bladder (6 ml h^−1^) as the EEG cycled between activated and slow wave sleep-like states. Note increased initial intervoid interval (arrow) on transition from activated to slow wave activity.

In the 14 rats in which urodynamic measurements were made during cyclical changes in EEG brain state, voiding became more irregular during SWA compared to the ‘activated’ state. Although when the data from all rats were grouped the mean inter-void interval did not change significantly between brain states (Table 1), for each rat the length of inter-void intervals displayed a greater range (SWA: 512 ± 61 s, activated: 336 ± 55 s, *p* < 0.01, paired t-test) during SWA compared to the ‘activated’ EEG state. Distinct changes in other urodynamic parameters also became apparent as the preparations cycled between different sleep-like brain states. During SWA, bladder compliance at the onset of the filling phase, i.e. immediately following a void, was significantly lower than in the “activated” state (Fig 3). This difference had become more pronounced by the end of the filling phase. Voiding threshold pressure was also higher during SWA compared to the “activated” EEG state but the detrusor contraction pressure (pressure rise prior to urine expulsion), and void pressure (bladder pressure during urine expulsion) were reduced (Fig. 1, Fig. 4 and Table 1). There was no significant change in the mean volume of urine expelled per void during different brain states (Table 1). Typically however, the transition from activated to SWA was characterized by an initial long inter-void interval (Fig 4). Since the bladder continued to fill with saline during this time, the bladder volume at which the next void was triggered was higher than in the activated brain state (mean increase 0.41 ± 0.22 ml). Moreover, the void volume threshold remained raised until the EEG cycled back to the activated state (Fig 4).

Changes were also observed in the characteristics of the EMG activity of the EUS during voiding in different brain states. During SWA the duration of the bursting EUS EMG activity during each void was shorter and the number of discrete bursting episodes during a void was smaller compared to the activated sleep-like state (Table 1).

### Single-unit activity in the PAG

**Fig. 5:**
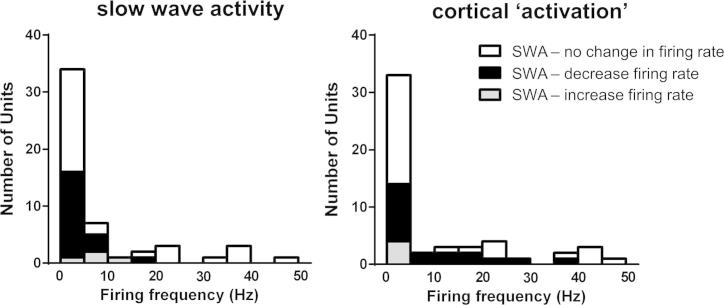
Distribution of firing frequencies of units recorded respectively during the upper and lower quartiles of EEG power at 0.5–1.5 Hz during cortical activation and slow wave activity.

**Fig. 6:**
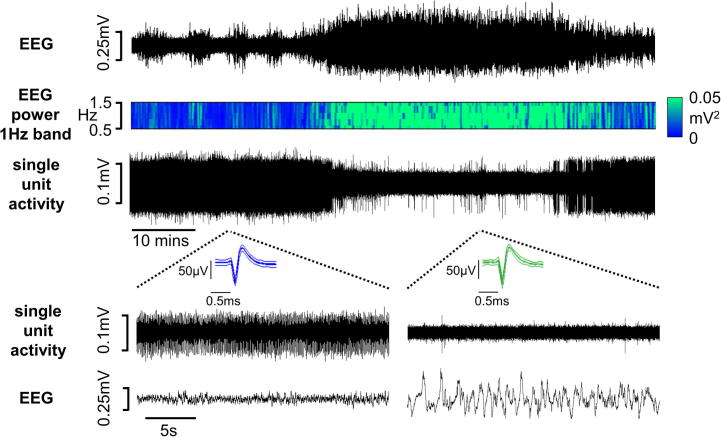
Change in spontaneous activity of a unit in the PAG during transition from activated to slow wave EEG state. Expanded excerpts of recordings show activity in more detail.

Spontaneous activity recorded from 52 units in the PAG or adjacent midbrain area showed a wide range of firing frequencies. For example, during SWA the firing rate of individual units ranged between 0.04 and 47 Hz (Fig 5). When the mean firing rate of individual cells was compared between the top and bottom quartiles of slow wave EEG power (0.5–1.5 Hz) during 1–3 cyclical changes in EEG brain state, the rate of ongoing activity was linked to EEG status in almost half of the cells (*n* = 23; 44.2%). In the majority of these (92%, *n* = 19) spontaneous activity decreased on transition from ‘activated’ state to SWA (activated: 9.9 ± 10.7 Hz, SWA: 2.6 ± 4.7 Hz) (Fig 6). These cells displayed a strong and statistically significant negative correlation between their spontaneous firing rate and slow wave EEG power (Spearman’s *r* = −0.73 ± 0.028, *p* < 0.001). A minority (4 cells, 7.7%) increased their firing rate during SWA (activated: 2.3 ± 1.5 Hz, slow wave: 6.9 ± 5.9 Hz), and displayed positive correlation between spontaneous firing rate and slow wave EEG power (Spearman’s *r* = 0.65 ± 0.040, *p* < 0.001). The remaining 29 units (55.8%) did not change their firing rate as the EEG brain state changed (9.3 ± 13.5 Hz during SWA *vs.* 9.4 ± 14.5 Hz in the ‘activated’ state) (Fig 5); little or no correlation was observed between spontaneous firing rate and slow wave EEG power (Spearman’s *r* = -0.11 ± 0.037).

**Fig. 7:**
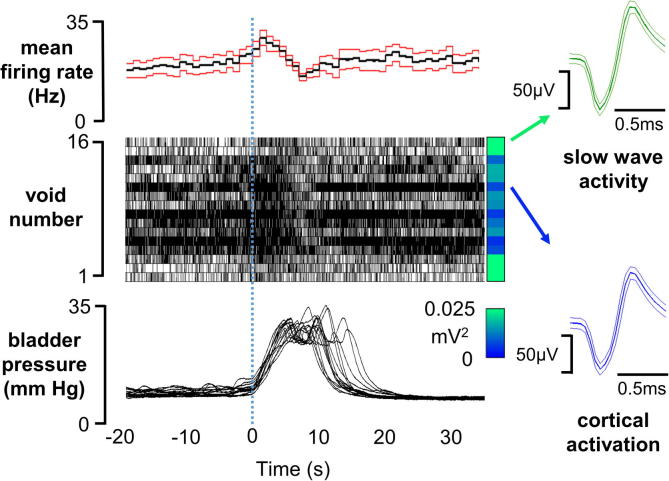
Activity of a micturition-responsive neuron in the PAG during change in brain activation state. Top trace: mean firing rate ± SEM (red lines) over 16 voids. Middle record: raster plot showing timing of spikes associated with each void. Vertical panel to the right shows color coding of EEG power spectrum for each void) brain state bottom record: overlay of change in bladder pressure during each void. Traces were time-locked to the onset of rise in bladder pressure. Note lower baseline activity and weaker micturition-linked activation during slow wave activity (period of high power in 1–1.5 Hz band, green color in EEG power spectrum) compared to activated EEG state (blue color in EEG power spectrum).

Almost a quarter of the cells recorded from (12/52, 23%) showed phasic changes in firing rate that were linked to the occurrence of voids (Fig 7). Of these, the activity of six cells was inhibited during voiding while firing rate increased in four cells; two further cells displayed a biphasic response (increase followed by decrease in activity) during voids. In nine of the 12 responsive cells (75%) the responses were reduced or abolished during SWS; and the response of a further cell was abolished during the ‘activated’ sleep-like state. The responses of two cells were unaffected by changes in sleep-like state. Interestingly, within the micturition-responsive population the basal firing rate of the majority (10/12, 83%) was sensitive to changes in EEG state. In contrast only 34% of non micturition-responsive cells (13/40) showed changes in firing rate that were associated with EEG state.

### Location of responsive cells

**Fig. 8:**
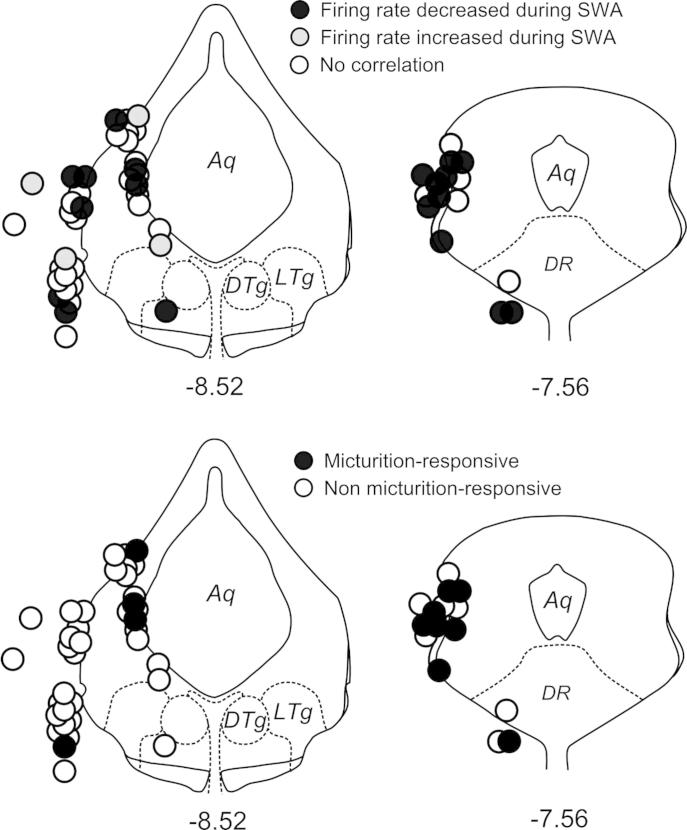
Top half: location of recording sites of spontaneously active neurons in the PAG and adjacent tegmentum during changes in EEG brain state. Bottom half. Location of micturition responsive cells. Recording sites plotted onto representative sections through the midbrain taken from the atlas of Paxinos and Watson (2007). Abbreviations. Aq, aqueduct; DR, dorsal raphe nucleus; DTg, dorsal tegmental nucleus; LTg, lateral tegmental nucleus. Numbers below sections indicate distance in mm caudal to Bregma.

Cells with ongoing activity linked to EEG state were intermingled with non-responsive cells. However, the activity of a higher proportion of cells in the PAG correlated with EEG status compared to cells located in the adjacent tegmental area (Fig 8). Similarly, the majority of micturition-responsive cells were located in the PAG rather than the adjacent area (Fig 8).

## Discussion

Among anesthetics that are commonly used for physiological experiments, urethane is unique in that it produces loss of consciousness and absence of responsiveness to painful stimuli, yet the EEG shows cyclical changes in waveform characteristic of natural sleep (Koyama et al., 1998, Imada et al., 2000, Clement et al., 2008, Pagliardini et al., 2012). In rats urethane is metabolized extremely slowly and exhaled as CO_2_. Pharmacokinetic studies have shown that even 8 h after i.v. administration of a near anesthetic dose, less than 20% had been exhaled (Nomeir et al., 1989). As a consequence, a single dose of urethane produces a long-lasting stable anesthesia. In the present study, the EEG waveform cycled between low-amplitude high-frequency desynchronized activity (‘activated’ state) passing through intermediate stages to high-amplitude synchronized SWA characteristic of slow wave sleep (‘deactivated’ state). The spectral characteristics during activated and deactivated states were similar to those observed previously in forebrain EEG during natural sleep and urethane anesthesia (Clement et al., 2008), although the total power was lower in our experiments due to extradural versus intracortical electrode placements. The transition between sleep-like states was associated with changes in respiratory activity: during the activated sleep-like state respiration was more rapid than during SWA and there was an increased incidence of augmented breaths as described previously (Pagliardini et al., 2012). However, we did not observe the rise in blood pressure on transition to the activated state reported by others (Clement et al., 2008), on the contrary, a small decrease in blood pressure was observed upon transition to the activated state.

In the present study urodynamic measurements made during repeated voids induced by continuous infusion of saline into the bladder were comparable to values reported in naturally sleeping animals (Kiddoo et al., 2006). In addition, we found that significant changes occurred as rats cycled between different sleep-like brain states. The threshold for micturition appeared to reset to a higher level during SWA. As the EEG cycled from high-amplitude SWA to the low-amplitude, desynchronized state there was a decrease in the threshold bladder pressure for voiding and bladder capacity (voiding threshold volume) while the peak bladder pressure achieved during voids and the bladder compliance increased. The transition from slow wave to desynchronized sleep is known to be associated with a unique re-patterning of sympathetic activity (Coote, 1982, Yoshimoto et al., 2011). Whether this affects control of bladder function is not known; similarly, little is known about how sleep states impact on responsiveness to afferent signals from internal organs. It is possible that during sleep there is a change in the level of the tonic descending inhibition on visceral afferent information at spinal levels (Ness and Gebhart, 1987). Alternatively or in addition, the intrinsic responsiveness to afferent input of the central circuitry controlling micturition may change with sleep state.

Micturition is dependent on the functional integrity of a spino-midbrain-spinal loop, which relays in the caudal ventrolateral PAG (Stone et al., 2011). In the present study the spontaneous activity of almost half of the neurons sampled in this area was influenced by the EEG brain state, with the majority of responsive cells showing an overall decrease in spontaneous activity during SWA compared to the ‘activated’ brain state. Importantly, the brain state-dependent cell population included micturition-responsive neurons, in which phasic changes in firing were time-locked to voids. The responses of the micturition-sensitive cells were reduced or in some cases completely abolished during SWA, indicating that the excitability of the micturition circuitry was depressed, in line with the increased threshold for voiding during SWA compared to the activated sleep-like state.

The midbrain micturition circuit is subject to a tonic inhibitory GABAergic influence that normally limits its functional excitability (Stone et al., 2011). The inhibition appears to be ‘lifted’ as voiding occurs (Kitta et al., 2008). The origin of the inhibitory tone on the PAG is not established but is likely to originate, at least in part, from frontal cortical areas. The prefrontal cortex sends direct projections to the PAG in rats as well as in primates (An et al., 1998, Floyd et al., 2000), which in the context of micturition, appear to be inhibitory (Nishijima et al., 2012). In the awake state the prefrontal cortex is thought to be involved in making high-level social judgments, such as behavioral inhibition and assessment of complex social cues (Damasio, 1996, Adolphs, 1999). The decision to “go” or “don’t go now” based on the social situation may be effected by modulating the level of excitability in the PAG micturition circuitry (Griffiths and Fowler, 2013, Lovick, 2015). Imaging studies in humans have shown that within the frontal lobes, the prefrontal cortex shows the greatest level of deactivation during sleep states (Braun et al., 1997). Although it is not possible to extrapolate from changes in fMRI BOLD signals to functional excitability at the neuronal circuit level, the imaging data are consistent with a top-down influence on the excitability of the midbrain micturition circuitry during sleep, which may vary with sleep state.

In humans and in rats too, fullness of the bladder is usually sufficient to induce arousal, which alerts the individual of the need to void (Kiddoo et al., 2006). In the present study in urethane-anesthetized rats, increases in bladder pressure that approached the voiding threshold did not induce electrocortical arousal. A small minority of voids during SWA were associated with transient (around 6 s) EEG “micro-arousals” as bladder pressure rose steeply at the start of a void but rising bladder pressure never evoked a transition from SWA to ‘activated’ EEG state or *vice versa.* This contrasts with previous reports of electrocortical arousal evoked in response to raising bladder pressure in urethane anesthetized rats (Page et al., 1992, Koyama et al., 1998). However, in those studies the bladder was inflated rapidly and pressures needed to evoke an arousal response (>70 mmHg) were well above the maximum values recorded in the present study, or indeed those likely to be encountered during physiological voiding, and may therefore represent a response to nociceptor activation.

The ability of physiological levels of bladder afferent activity to induce micturition while failing to evoke arousal is a feature of nocturnal enuresis in humans. This common problem has been estimated to afflict 1–2% of over 15 year olds (Kiddoo, 2011). The relationship between wetting and EEG sleep state is not clear. Some studies report that wetting occurs more often during ‘stage 4’ or ‘slow wave sleep’ and rarely during REM sleep (Pierce et al., 1961, Ritvo et al., 1969) but others have failed to detect a relationship (Mikkelsen et al., 1980, Inoue et al., 1987, Bader et al., 2002), while another study reported that wetting occurred primarily during periods of very deep sleep (Wolfish et al., 1997). In line with the latter findings a more recent study using sophisticated EEG power analysis in adolescents and adults with primary nocturnal enuresis revealed an abnormally deep sleep in enuretics who, when water loaded prior to sleeping, failed to be roused and voided while continuing to sleep (Hunsballe, 2000). If during exceptionally deep sleep, the prefrontal cortex becomes deactivated to an extent that not only is it no longer responsive to interoceptive signals from the bladder, but at the same time the tonic descending control it normally exerts on the micturition circuitry in the PAG also becomes markedly reduced or fails, micturition may proceed via the midbrain circuitry without interrupting sleep.

The present study, using continuous cystometry in urethane-anesthetized rats, has demonstrated that changes in urodynamic parameters, which correspond with changes in the excitability of the midbrain micturition circuitry in the PAG, occur during spontaneous transition between different sleep-like EEG brain states. Since under urethane micturition proceeds without evoking arousal, the preparation mimics nocturnal enuresis in humans and may prove to be of value for further investigation of this condition. The data also highlight the plastic nature of the PAG circuitry with respect to brain activation status. These findings may extend beyond micturition to impact on controls over other bodily systems and behaviors, which are integrated by the PAG.

## Figures and Tables

**Table 1:** Urodynamic variables during slow wave and ‘activated’ sleep-like states. Mean values ± SEM

	Void pressure threshold (mmHg)	End-fill compliance (ml/mmHg)	Start-fill compliance (ml/mmHg)	Void volume (ml)	Detrusor contraction pressure (mmHg)	Void pressure (mmHg)	Void interval (s)	EUS burst duration (s)	No. bursting episodes per void
Mean SWA	13.15 ± 0.55	0.11 ± 0.02	0.70 ± 0.13	0.65 ± 0.085	18.04 ± 1.54	26.61 ± 1.12	294 ± 29	5.39 ± 0.75	2.01 ± 0.34
Mean ‘activated’	10.79 ± 0.41	0.20 ± 0.02	1.04 ± 0.09	0.68 ± 0.10	22.26 ± 1.22	28.38 ± 1.09	273 ± 33	6.64 ± 0.81	2.51 ± 0.42
Mean difference	2.36 ± 0.41	−0.08 ± 0.02	−0.35 ± 0.12	−0.03 ± 0.067	−4.22 ± 0.58	−1.77 ± 0.41	21 ± 21	−1.26 ± 0.54	−0.50 ± 0.19
*p*	<0.001	<0.001	<0.01	>0.05	<0.001	<0.001	>0.05	<0.05	<0.05
Significance	∗∗∗	∗∗∗	∗∗	n.s.	∗∗∗	∗∗∗	n.s.	∗	∗
